# Complete resorption of the humerus in metastatic thyroid carcinoma: a case report

**DOI:** 10.1186/s12891-024-07250-2

**Published:** 2024-02-27

**Authors:** Xiajie Huang, Yeping Chen, Daofu Zeng, Rongyuan Liang, Zhidong Liao, Guizheng Wei, Wenjun Hao, William Lu, Yan Chen

**Affiliations:** 1 Department of Bone and Joint Surgery, The First Affiliated Hospital of Guangxi Medical University, Nanning, China; 2 Department of Orthopaedics and Traumatology, The University of Hong Kong, Hong Kong, China

**Keywords:** Thyroid carcinoma, Bone metastasis, Humeral metastasis, Surgical treatment, Individualized treatment, Multidisciplinary approach

## Abstract

**Background:**

Thyroid carcinoma is the most common endocrinological malignancy, but its spread to bone is rare. Particularly, bone metastases leading to complete resorption of the humerus are extremely uncommon. We aimed to explore factors affecting treatment decision in humeral metastasis by presenting a case and analyze the possible treatments via conducting a literature review.

**Case presentation:**

We described a case of a 68-year-old woman experiencing chronic pain in her right upper arm for six years. Clinical, radiological, and pathological evaluations confirmed humeral metastasis from thyroid carcinoma. Surgical treatments like tumor removal or limb amputation were suggested for prolonging life and pain relief, but the patient refused them and pursued conservative managements such as herbal medicine, radioactive iodine (^131^I) therapy, and Levothyroxine Sodium(L-T4). The humeral destruction aggravated gradually, ultimately leading to complete resorption of her right humerus. The patient could not move her right shoulder, but her forearm motion was almost normal; thus, she could complete most of her daily living activities independently. Surgical treatments such as limb amputation were advised but she still refused them for preservation of the residual limb function and preferred conservative managements.

**Conclusion:**

A personalized multidisciplinary approach is important for patients with bone metastasis. The balance between limb amputation for life-prolonging and pain relief and limb salvage for preservation of residual function and social and psychological well-being should be considered. Our literature review revealed that some novel surgical treatments and techniques are available for bone metastases. This case adds to our current understanding of bone metastases and will contribute to future research and treatments.

**Supplementary Information:**

The online version contains supplementary material available at 10.1186/s12891-024-07250-2.

## Background

Thyroid carcinoma is the most common endocrine cancer worldwide [1, 2]. Epidemiological studies demonstrated a significant increase in the incidence of thyroid carcinoma over the past two decades, particularly in Asian countries [3]. Distant metastases are rare in thyroid carcinomas, with bone involvement found in only 3.9 to 4.2% of cases [4, 5]. In such cases, the axial skeleton, particularly the spine and ribs, are commonly affected, leading to symptoms such as severe pain and compression fractures [6]. The complete resorption of the humerus as an initial presentation of late diagnosis of differentiated thyroid carcinoma (DTC) is extremely rare and has been scarcely reported.

Studies indicated a mortality rate of 67.2% within an 8-year follow-up for follicular thyroid cancer with bone metastasis [7]. One of the treatment challenges is the balance between extending the patient’s survival time and preserving the quality of life. Here, we described a case of a woman who experienced chronic pain in her right upper arm due to metastasis from thyroid carcinoma for six years, and then, we conducted a literature review to summarize the advancements in the treatment of bone metastasis related to thyroid carcinoma. We aimed to explore factors affecting treatment decision in humeral metastasis by presenting a case (a woman with complete resorption of humerus due to thyroid carcinoma) and analyze the possible treatments via conducting a literature review.

## Case presentation

**Fig. 1:**
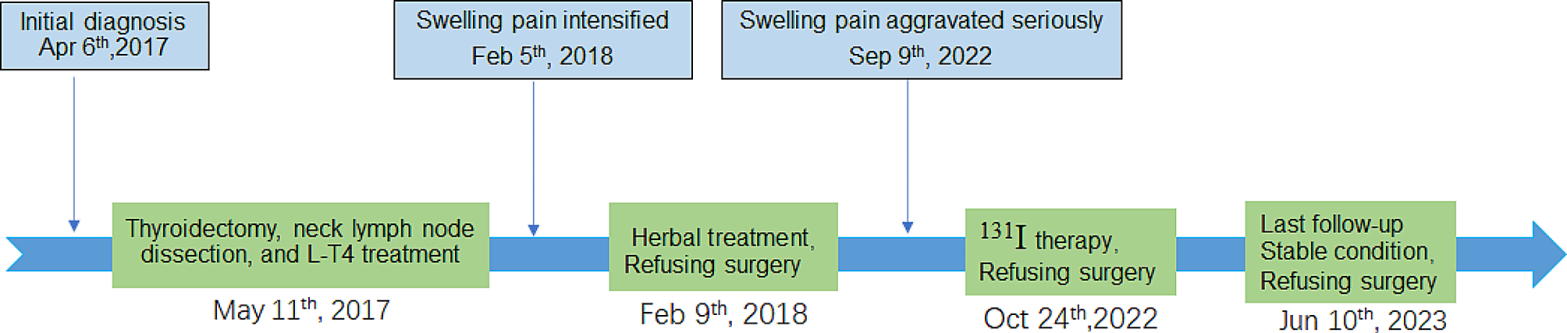
The timeline of the diagnosis and treatment course of the patient in the present case

**Fig. 2:**
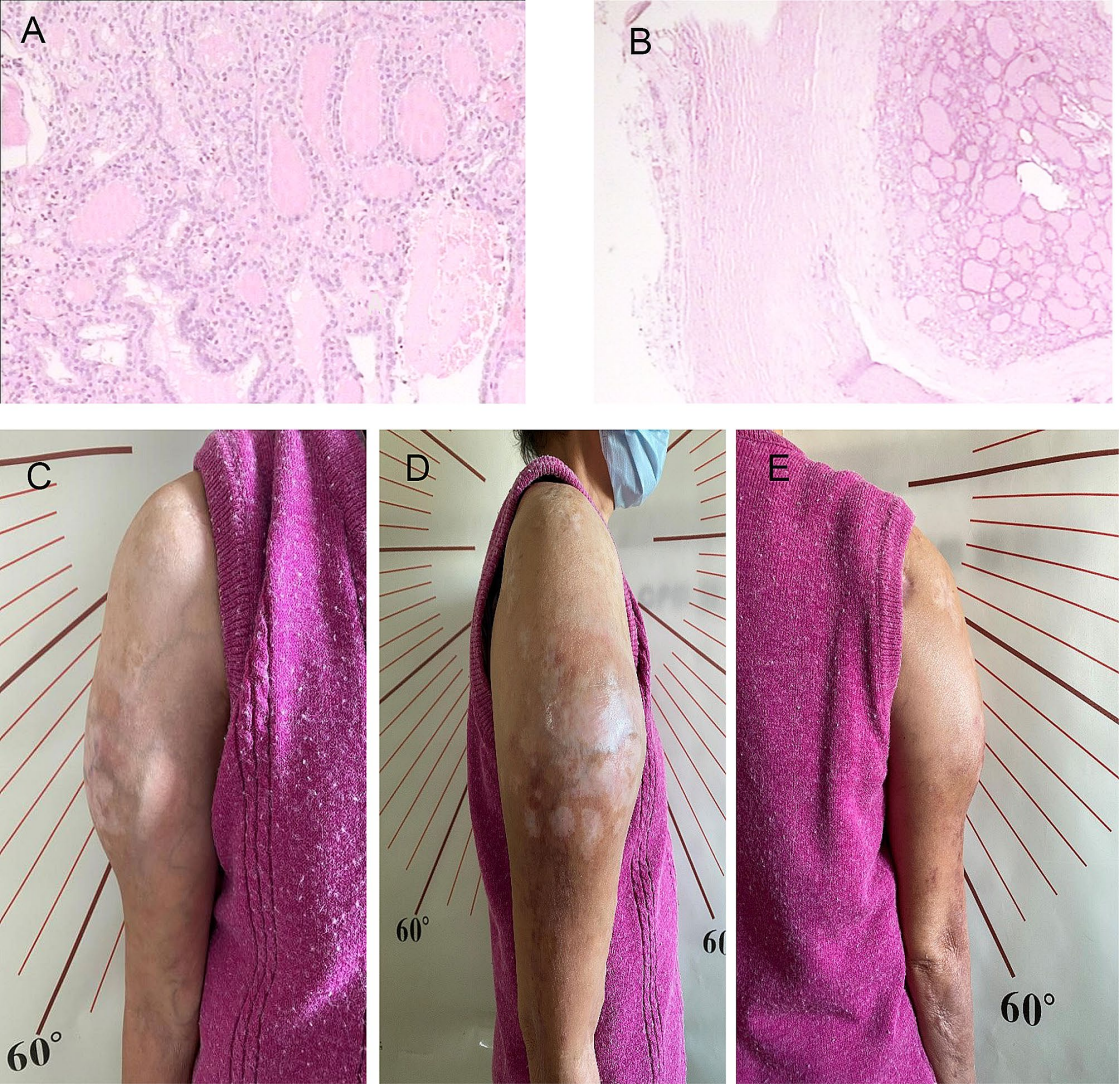
Pathological examination and measurement of the range of motion of the right upper arm. (**A**) A biopsy of the right humerus revealed bone metastases of follicular thyroid carcinoma. (**B**) Postoperative pathological examination confirmed a typical follicular thyroid carcinoma originating from the thyroid gland. (**C**) Anterior view. (**D**) Lateral view. (**E**) Posterior view

A 68-year-old woman was admitted to the Department of Nuclear Medicine of our hospital for intermittent chronic pain in her right upper arm, which had lasted six years and worsened over the past month, on September 9th, 2022, (Fig. 1). Six years earlier, the patient had acute pain in her right upper limb following a fall, although no skin injuries were evident. She did not undergo any imaging investigations and only sought treatment from a traditional healer who prescribed her some herbal medicine. Despite this treatment, the pain persisted for six months and progressively intensified. She gradually lost the mobility in the right shoulder and finally sought medical help. X-ray imaging revealed obvious erosion of the proximal humeral cortex and bone marrow, indicating lytic bone metastasis. A subsequent bone biopsy confirmed the diagnosis of bone metastasis from follicular thyroid carcinoma (Fig. 2A). Orthopedic specialists recommended surgical interventions such as tumor excision or upper limb amputation for prolonging life and pain relief. However, the patient declined any surgical intervention. She pursued alternative methods of care, including massage, stretching exercises, and herbal medicine during the subsequent six years. In the month preceding her admission, she had increased nocturnal pain in her upper limb, which subsided to some extent during daytime movement.

The patient’s medical history was characterized by diagnostic endeavors aimed at confirming the presence of humeral metastasis. These efforts encompassed a series of procedures, including computed tomography (CT) scan, ultrasound, magnetic resonance imaging (MRI), and needle biopsy of her right upper arm, all of which corroborated the thyroid as the primary cancer site. Following these diagnostics, the patient underwent partial thyroidectomy and modified lymph node dissection performed by a surgical team. Postoperative pathological examination confirmed follicular thyroid carcinoma, with evidence of capsular and lymphovascular infiltrations (Fig. 2B). Consequently, thyroid ablation was administered twice, two and six months postoperatively, respectively, reaching a total dose of ^131^I at 200 mCi. Furthermore, the patient received L-T4 treatment to maintain a thyroid stimulating hormone (TSH) level below 0.1 µIU/mL. Despite the utilization of herbal medicines and L-T4 administration twice daily for over a year, the patient experienced no significant pain relief. Moreover, the swelling in her right upper arm progressively worsened. In terms of personal and family history, the patient had not reported instances of food or drug allergies or genetic diseases.

During the physical examination, severe swelling in the middle and proximal upper arm was found (Fig. 2C, D, E). The patient’s sensory functions were generally fine, although numbness and tingling was present some parts of her right upper arm. Importantly, the ability to actively move her right shoulder was limited, affecting movements like bending and straightening, moving towards and away from her body, and rotating inward and outward (Supplementary video 1). On the contrary, her forearm displayed relatively normal movement, allowing her to bend and straighten her elbow, turn her palm up and down, move her wrist back and forth, and perform various finger movements such as bending, spreading, bringing together, and pushing back (Supplementary videos 2 and 3). Consequently, she could still manage most of her daily activities independently.

**Fig. 3:**
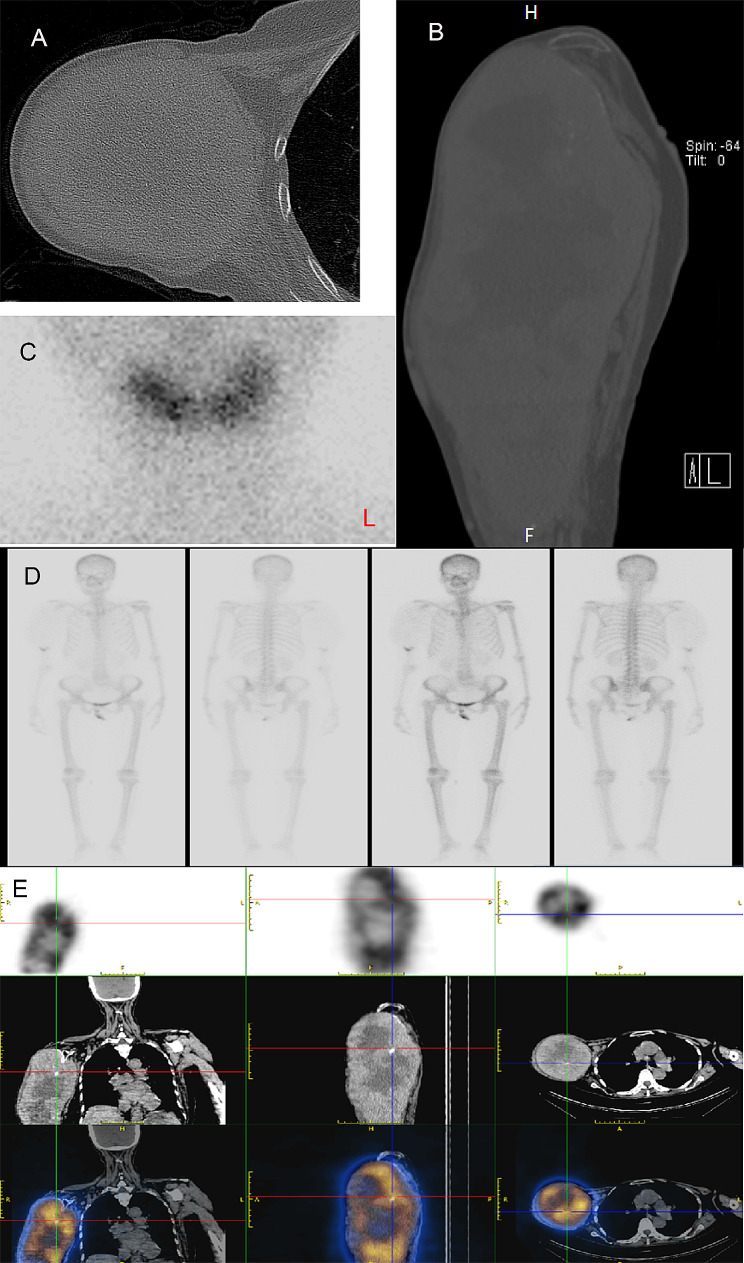
(**A, B**) Computed tomography (CT) images of the right shoulder and upper arm. (**C**) Thyroid emission computed tomography scan. (**D**) Whole-body bone imaging. (**E**) Whole-body imaging on the fifth day after oral administration of 350 mCi of 131I. There was no clear residual functional tissue in the thyroid region, but obvious functional metastasis could be seen in the right upper arm

Then, a series of imaging examinations were performed to further evaluate the patient’s condition. A CT scan showed complete absence of her upper humerus and metastases in the axillary lymph nodes and both lungs (Fig. 3A, B). Furthermore, a whole-body bone scan demonstrated lytic bone destruction in her right upper limb and thyroid carcinoma (Fig. 3D). Thyroid emission computed tomography (ECT) scans showed no noticeable absorption of the radioactive substance 99mTcO4 in the neck area (Fig. 3C). Ultimately, the patient was diagnosed with follicular thyroid carcinoma with metastasis to her right humerus and both lungs.

Upon consultation with the surgical team, she was advised to undergo upper limb amputation. However, the patient declined it since the functionality of her right hand remained intact. Due to significant swelling and tenderness in the affected area, along with the identification of large fluid-filled regions on both the CT scan and MRI, the patient received ^131^I treatment without any notable discomfort (Fig. 3E). Subsequent therapeutic options would be contemplated based on the evolving clinical situation. A follow-up plan included a recommendation for the patient to visit the orthopedic department approximately 3 to 4 weeks post-discharge for local fluid aspiration.

**Fig. 4:**
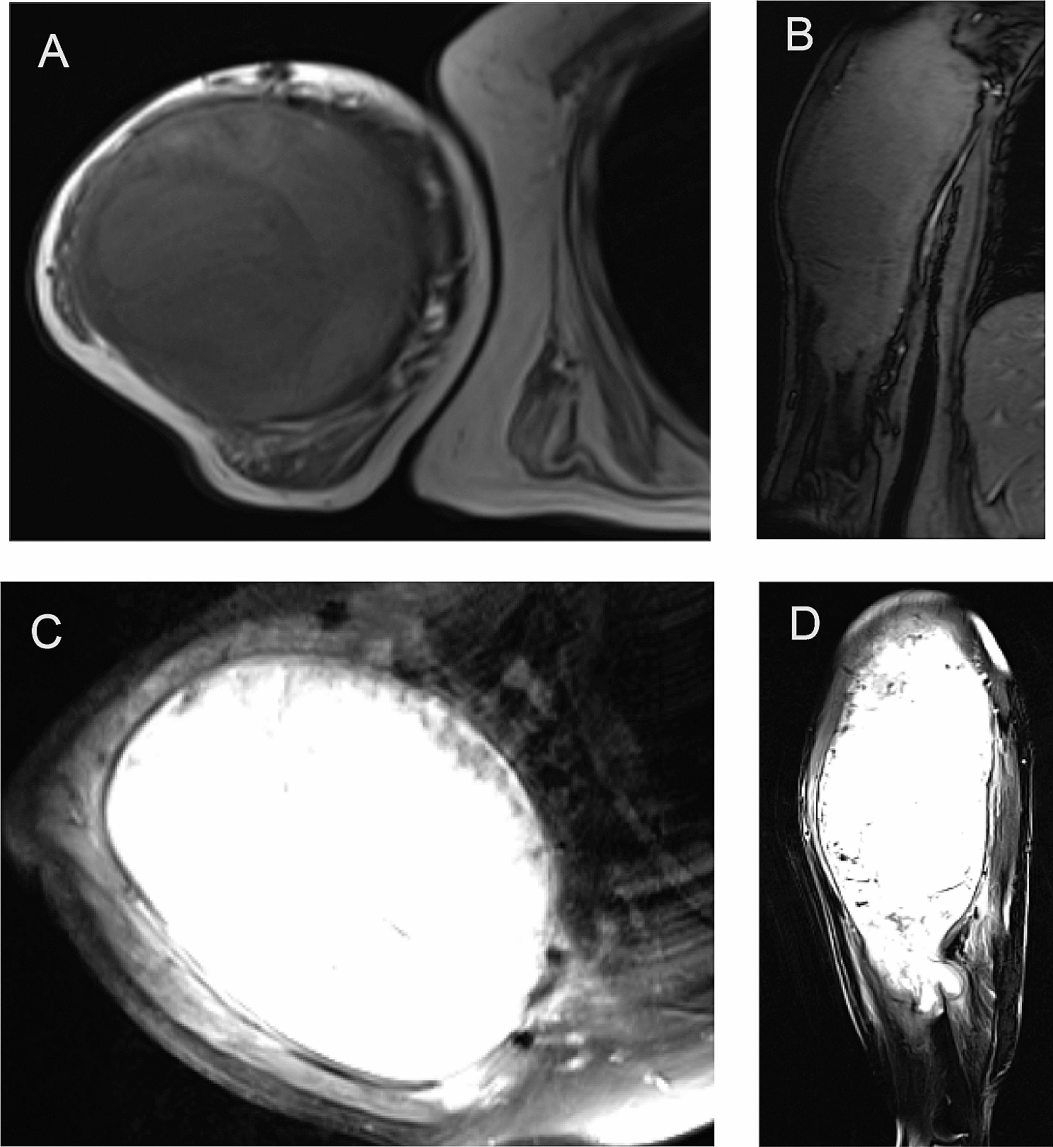
(**A, B, C, D**) Magnetic resonance imaging (MRI) of the right shoulder and the right upper arm

In June 2023, physical examination showed that the patient’s general condition remained stable, and the mobility of her right forearm exhibited no substantial alterations relative to previous assessments (Supplementary videos 4–5). Furthermore, MRI examination of the patient’s right shoulder and right upper arm indicated no significant progression compared to earlier results (Fig. 4). The patient refused against any surgical intervention for her right upper arm.

## Literature review

A literature review was conducted using the three major databases: PubMed (https://pubmed.ncbi.nlm.nih.gov/), Web of Science (https://www.webofscience.com/wos/woscc/basic-search), and EBSCOhost (https://search.ebscohost.com/), to retrieve English articles reporting on bone metastasis in follicular thyroid carcinoma from January 2003 to July 2023. The search terms include “follicular thyroid carcinoma”, “thyroid cancer”, “bone”, “Bone metastases”, and “case report”. We included only papers that were published in English and had the full text available.

A total of 21 articles reporting on 22 cases were obtained (Table 1) [8–27]. All cases were confirmed to have follicular thyroid carcinoma with bone metastasis. The average age of the 22 patients was 60.0 ± 11.2 years, with a prominent female predilection (male-to-female ratio of approximately 1:4). Eighteen patients underwent total/near-total thyroidectomy or lobectomy, resulting in effective control of the primary lesion, indicating the efficacy of primary tumor management strategies. The most common sites of metastasis were the skull (36.4%, 8/22) and spine (31.8%, 7/22), while other sites such as the mandible (13.6%, 3/22), ribs (9.0%, 2/22), femoral head (4.5%, 1/22), hip bone (4.5%, 1/22), and humerus (4.5%, 1/22) were also reported. The tendency of metastasis to certain bones, particularly the skull and spine, is a key area of concern. This contributes to diverse symptoms seen in patients. Common symptoms such as pain, swelling, and limb weakness, along with specific issues like numbness and limb weakness in cases of spinal metastasis, highlight the clinical challenges linked to bone involvement in follicular thyroid carcinoma. Intervention strategies for bone metastasis included adjuvant therapy with ^131^I (68.2%, 15/22), surgical resection (50.0%, 11/22), suppression therapy with thyroid hormones (40.9%, 9/22), and chemotherapy (4.5%, 1/22). Surgery is still the main method for treating bone metastases, among the varied interventions available (surgical resection, reconstruction, adjuvant therapies, and chemotherapy) [28]. Among the 11 surgical cases, orthopedic procedures made up 90.9%, involving activities such as joint replacement or fusion(45.5%, 5/11), prosthesis reconstruction(18.2%, 2/11), internal fixation(18.2%, 2/11), bone cement formation(9.1%, 1/11), and decompression surgery(9.1%, 1/11). With the ongoing progress and expansion of minimally invasive surgical techniques, the effectiveness of surgical treatment may continue to improve in the future [29]. Furthermore, the comprehensive approach to handling bone metastasis emphasizes the need for a personalized and multidisciplinary therapy. The wide range of interventions, including surgery, reconstruction, and additional therapies like ^131^I and thyroid hormone suppression, highlights the difficulty in standardizing treatment methods. Notably, the significant reliance on therapies like ^131^I underscores its role in managing bone metastasis, though it is often used alongside other treatments. However, the limited use of chemotherapy in addressing bone metastasis underscores the ongoing search for more effective overall treatments. The relatively low use may be due to concerns about effectiveness, disease-specific limitations, or a preference for localized treatments.

**Table 1:** Clinical data of 22 cases with bone metastasis from follicular thyroid carcinoma

Authors	Year	Age (year)	Sex	Symptom of metastasis	Location of metastasis	Surgery on primary site	Treatments of bone metastasis	Outcomes
Akdemir et al. [9]	2005	57	M	Headache and a mass in the right occipitoparietal region	Skull	Total thyroidectomy	Metastasectomy, radiotherapy	No recurrence for 12 months
Araki et al. [10]	2008	55	F	The feeling of constant discoordination after tooth extraction and gingival swelling	Mandible	Total thyroidectomy	Mastectomy, radiotherapy	Not available
Matsuno et al. [8] Present Case 1	2010	58	F	Hearing disturbance and swelling of the right maxilla	Skull base	Left thyroidectomy	Metastasectomy, ^131^I therapy and thyroid hormone	No recurrence for 12 months
Present Case 2		71	F	Hoarseness	Skull base	Right thyroidectomy	Metastasectomy, ^131^I therapy and thyroid hormone	No recurrence for 12 months
Sandu et al. [11]	2011	50	F	Progressive spinal cord compression	Vertebra	Total thyroidectomy	Metastasectomy, bone cement plaster, posterior arthrodesis and ^131^I therapy	No recurrence for 36 months
Tahamtan et al. [12]	2012	42	F	Frontal scalp subcutaneous nodule	Skull	Total thyroidectomy	Reconstruction of bone defect	Not available
Koppad et al. [13]	2012	48	F	Swelling in the frontal region of the skull	Massive skull	No intervention	Supraphysiological, dose of thyroxine	Not available
Coban et al. [14]	2014	63	F	Bowel and bladder incontinence	Vertebra	Total thyroidectomy	^131^I therapy	Not available
C et al. [15]	2014	76	M	Painless swelling in the left side body of the mandible	Mandible	Not available	Not available	Not available
De Pasquale et al [16].	2014	65	F	A large painful mass developed on the left inferior part of her face	Mandible	Total thyroidectomy	Metastasectomy, ^131^I therapy, prosthetic bone reconstruction	No recurrence for 46 months
Zhang et al. [17]	2014	65	F	Progressive swelling of her neck	Cervical vertebra	Total thyroidectomy	Metastasectomy, internal fixation, ^131^I therapy, and thyroid hormone	No recurrence for 15 months
Mizoshiri et al. [18]	2015	68	F	Left hip joint pain	Femoral head; right rib	Partial thyroidectomy	Bipolar hip arthroplasty, metastasectomy	No recurrence for 18 months
Bansal et al. [19]	2016	55	F	Progressively increasing swelling on the scalp	Skull	Total thyroidectomy	No intervention	No recurrence for 6 months
Baião et al. [20]	2017	84	F	Acute paraparesis	Vertebra	Total thyroidectomy	^131^I therapy and thyroid hormone	No recurrence for 6 months
Akgedik et al. [21]	2017	51	M	Pleuritic pain	Rib	Total thyroidectomy	Metastasectomy, ^131^I therapy, and thyroid hormone	No recurrence for 12 months
Yang et al. [30]	2017	38	M	Pathological fracture of the humerus	Humerus	Total thyroidectomy	Metastasectomy, replacement of left proximal humerus, and ^131^I therapy	Not available
Karimifar et al. [22]	2018	52	M	Hip fracture and hypercalcemia	Hip and vertebra	Total thyroidectomy	Metastasectomy, internal fixation, ^131^I therapy, and thyroid hormone	Not available
Liu et al. [23]	2021	65	F	Numbness in right hand, weakness in right lower limbs	Skull	Not available	Metastasectomy	Not available
Omar et al. [24]	2022	74	F	Sacral pain	Sacral region	Right thyroidectomy	Decompressive surgery, ^131^I therapy, and thyroid hormone	Not available
Yang et al. [25]	2022	57	F	Progressive dysphagia, hoarse voice, weakness of her right shoulder, and a goiter.	Skull base	Total thyroidectomy	Radiotherapy, chemotherapy	Not available
Chen et al. [26]	2022	67	F	Vertebral pain	Vertebra	Total thyroidectomy	Artificial vertebral body replacement, ^131^I therapy, and thyroid hormone	No recurrence for 12 months
Fataftah et al. [27]	2022	60	F	Knee pain and limping	Knee	Total thyroidectomy	Total knee replacement, ^131^I therapy	No recurrence for 12 months

## Discussion and conclusion

Follicular thyroid carcinoma is the second most common differentiated type of thyroid cancer, accounting for approximately 10–15% of differentiated thyroid cancers [31]. Typical sites of bone metastasis in follicular thyroid carcinoma include the skull, spine, mandible, and ribs [6]. Follicular thyroid carcinoma spreading to humerus is extremely rare, with only one case reported in the past 20 years [30]. The presented case underscores the challenging in decision-making process in managing humeral metastasis from thyroid carcinoma, especially when the humerus was completely.

When weighing surgical options, the choice between limb amputation and limb salvage is not always easy. The potential life-saving benefits of limb amputation must be balanced against the functional advantages of limb salvage [7]. This highlights the need for personalized treatments, considering the patient’s medical history, preferences, and values. Furthermore, our case underscores the importance of multidisciplinary treatment in managing rare and complex bone metastasis.

In conclusion, our case contributes to medical literature, emphasizing the importance of personalized and multidisciplinary approach for patients with bone metastasis. This patient-centered approach, tailored to the unique circumstances of rare cases, is crucial for optimal outcomes and advancing our understanding of managing humeral metastasis from thyroid carcinoma.

### Electronic supplementary material

Below is the link to the electronic supplementary material.

Supplementary video 1

Supplementary video 2

Supplementary video 3

Supplementary video 4

Supplementary video 5

Supplementary Material 1

## Data Availability

Data is provided within the manuscript or supplementary information files.

## Abbreviations

^131^I: Radioactive iodine
L-T4: Levothyroxine Sodium
DTC: Differentiated thyroid carcinoma
CT: Computed tomography
MRI: Magnetic resonance imaging
TSH: Thyroid stimulating hormone
