# Supplementary material for: Complete resorption of the humerus in metastatic thyroid carcinoma: a case report
Source: BMC Musculoskelet Disord. 2024 Feb 27;25:177. doi: 10.1186/s12891-024-07250-2 (PMC10897982; doi:10.1186/s12891-024-07250-2)
Supplement: Supplementary file 6 — Supplementary Material 1 [file 12891_2024_7250_MOESM6_ESM.pdf]

CARE Checklist of information to include when writing a case report

CARE case report guidelines

(cc) BY-NC-ND

Topic	Item	Checklist item description	Reported on Line
**Title**	1	The diagnosis or intervention of primary focus followed by the words "case report"	Complete resorption of the humerus in metastatic follicular thyroid carcinoma: A case report and literature review.
**Key Words**	2	2 to 5 key words that identify diagnoses or interventions in this case report, including "case report" . . .	case report, humerus, follicular thyroid carcinoma, bone metastasis, individualized treatment.
**Abstract (no references)**	3a	Introduction: What is unique about this case and what does it add to the scientific literature?	The case describes a rare occurrence of complete resorption of the humerus in metastatic follicular thyroid carcinoma, emphasizing the challenges in diagnosis and management.
	3b	Main symptoms and/or important clinical findings	Intermittent chronic pain and huge swelling of the right upper arm
	3c	The main diagnoses, therapeutic interventions, and outcomes	Diagnosed with bone metastasis from follicular thyroid carcinoma, declined surgical interventions and opted for herbal medicine, radioactive iodine (131I) therapy, and Levothyroxine Sodium (L-T4).
	3d	Conclusion—What is the main "take-away" lesson(s) from this case?	Individualized and multidisciplinary treatment approaches are crucial in managing rare presentations like complete humeral resorption in metastatic follicular thyroid carcinoma.
**Introduction**	4	One or two paragraphs summarizing why this case is unique (**may include references**)	The case presented here showcases a highly unusual occurrence of complete resorption of the humerus due to metastatic follicular thyroid carcinoma, which is an exceedingly rare manifestation in the context of thyroid carcinomas. Typically, bone involvement in thyroid carcinomas is infrequent, reported in only about 3.9-4.2% of cases, and commonly affects axial skeleton areas like the spine and ribs. However, the total resorption of the humerus as an initial presentation of late-diagnosed differentiated thyroid carcinoma is a remarkably rare and scarcely documented occurrence in medical literature. The rarity of this case emphasizes the challenges in both diagnosing and effectively managing bone metastasis in the context of follicular thyroid carcinoma. Furthermore, the patient's refusal of surgical intervention and their choice to opt for conservative management through herbal medicine, radioactive iodine therapy, and Levothyroxine Sodium (L-T4) adds a unique dimension to the management strategies, highlighting the complexities and individualized nature of treating such atypical manifestations of thyroid cancer.<br><br>This unique case emphasizes the significance of tailored, multidisciplinary, and personalized treatment approaches in managing rare presentations such as complete humeral resorption in metastatic follicular thyroid carcinoma. By underscoring the challenges in both diagnosis and treatment, the case sheds light on the need for a comprehensive approach, incorporating individual patient preferences while weighing the potential risks and benefits of different treatment modalities. Moreover, the extensive literature review conducted in this study offers valuable insights into the rare presentation of complete humeral resorption due to follicular thyroid carcinoma, contributing to the existing body of knowledge. However, the limited number of cases underscores the necessity for further research and more extensive studies to establish standardized treatment protocols, thereby enhancing the prognosis for patients confronted with such unusual manifestations of thyroid cancer.
**Patient Information**	5a	De-identified patient specific information.	A 67-year-old woman with a five-year history of chronic pain in the right upper arm.

5b Primary concerns and symptoms of the patient. . . . . Intermittent chronic pain in the right upper arm and increased swelling and pain in the right upper arm.

5c Medical, family, and psycho-social history including relevant genetic information . . . . No reported food or drug allergies, or genetic diseases in personal and family history.

5d Relevant past interventions withoutcomes . . . . Multiple diagnostic procedures confirmed the primary site as the thyroid gland and partial thyroidectomy was performed.

Clinical Findings

6 Describe significant physical examination (PE) and important clinical findings. . . . . Severe swelling in the upper arm, limitations in shoulder movement, but unimpaired forearm motion.

Timeline

7 Historical and current information from this episode of care organized as a timeline . . . . Detailed description covering historical information and the current case progression over five years.

Diagnostic Assessment

8a Diagnostic testing (such as PE, laboratory testing, imaging, surveys). . . . . CT scan, ultrasound, MRI,bone biopsy, and diagnostic interventions to confirm thyroid as the primary site of cancer.

8b Diagnostic challenges (such as access to testing, financial, or cultural) . . . . Low cancer awareness, delayed diagnosis, and challenges in treating metastatic thyroid carcinoma.

8c Diagnosis (including other diagnoses considered) . . . . Diagnosed with follicular thyroid carcinoma with bone metastasis in the humerus and both lungs.

8d Prognosis (such as staging in oncology) where applicable . . . . Follow-up at 6 months, the magnetic resonance imaging (MRI) of the patient's right shoulder joint and upper right arm showed no significant progression compared to earlier results. Additionally, the patient's overall condition remained stable, and there were no notable changes in the mobility of the right forearm compared to the previous assessment.

Therapeutic Intervention

9a Types of therapeutic intervention (such as pharmacologic, surgical, preventive, self-care) . . . . Conservative management with herbal medicine, radioactive iodine (131I) therapy, and Levothyroxine Sodium (L-T4) treatment.

9b Administration of therapeutic intervention (such as dosage, strength, duration) . . . . Administered L-T4 twice daily for over a year to address the metastatic lesion in the right humerus.

9c Changes in therapeutic intervention (with rationale) . . . . The patient 's condition was stable and there was no significant progress.

Follow-up and Outcomes

10a Clinician and patient-assessed outcomes (if available) . . . . There was no significant progress in physical examination and MRI examination after 6 months of follow-up.

10b Important follow-up diagnostic and other test results . . . . There was no significant progress in physical examination and MRI examination.

10c Intervention adherence and tolerability (How was this assessed?) . . . . Patient declined surgical interventions.

10d Adverse and unanticipated events . . . . No specific adverse events reported.

Discussion

11a A scientific discussion of the strengths AND limitations associated with this case report . . . . Highlights the importance of individualized and multidisciplinary treatment approaches in managing rare presentations.

11b Discussion of the relevant medical literature **with references**. . . . . Provides a comprehensive review of relevant case reports related to bone metastasis in follicular thyroid carcinoma.

11c The scientific rationale for any conclusions (including assessment of possible causes) . . . . Emphasizes the challenges and complexities in managing bone metastasis in rare forms of thyroid cancer and the importance of personalized treatment.

11d The primary "take-away" lessons of this case report (without references) in a one paragraph conclusion . . . . Individualized and multidisciplinary treatment is crucial in managing rare presentations.

Patient Perspective

12 The patient should share their perspective in one to two paragraphs on the treatment(s) they received . . . . Patient opted for conservative treatments despite surgical recommendations, showing a preference for alternative methods of care.

Informed Consent

13 Did the patient give informed consent? Please provide if requested . . . . **Yes** √ **No** ☐
